# Co/Mo bimetallic addition to electrolytic manganese dioxide for oxygen generation in acid medium

**DOI:** 10.1038/srep15208

**Published:** 2015-10-15

**Authors:** Dario Delgado, Manickam Minakshi, Justin McGinnity, Dong-Jin Kim

**Affiliations:** 1 School of Engineering and Information Technology, Murdoch University, Murdoch, Australia; 2 Mineral Resources Research Division, Korea Institute of Geoscience and Mineral Resources, Daejeon, South Korea

## Abstract

An efficient electrocatalyst comprising inexpensive and earth-abundant materials for the oxygen evolution reaction (OER) is crucial for the development of water electrolysis. In this work, *in-situ* addition of cobalt/molybdenum ions to the electrolytic manganese dioxide has been shown to be beneficial for the OER in acid solution as its overpotential performed better (305 mV) than that of the commercial DSA^®^ (341 mV) at 100 mA cm^−2^. The OER was investigated at ambient temperature in 2 M H_2_SO_4_ solution on the modified EMD (MnMoCoO) electrodes. The energy efficiency of the MnMoCoO electrodes improved significantly with the amount of Co in the plating solution. For the electrodeposited catalysts, physico-chemical and electrochemical measurements were conducted including static overpotentials. The better performance of the modified EMD was attributed to an improved charge transfer resistance (*R*_ct_; 0.290 Ω cm^2^), average roughness factor (*r*_*f*_; 429) and decrease in water content in the electrodeposited catalysts. The kinetic parameters obtained on MnMoCoO catalysts were compared and discussed according to the cobalt concentration.

Hydrogen is an abundant, renewable and clean energy source and has been considered as a solution to the problems arising from the current unsustainable fossil fuel economy. Recently, the hydrogen economy is gaining the attention of government bodies and major oil companies^1^,^2^,^3^,^4^,^5^,^6^,^7^,^8^. Hydrogen can be generated through various methods including thermochemical, electrochemical and biochemical^9^. Of all these available methods, water electrolysis (via electrochemical) is suitable for large scale production of hydrogen. Electrolysis of water is a simple way to produce hydrogen with high purity at an economical price when a source of energy to drive the electrolysis is available such as solar or nuclear energy^1^.

The water electrolysis consists of two electrochemical reactions, termed as hydrogen and oxygen evolution reactions (HER and OER). The equilibrium potential of water electrolysis is 1.23 V at standard conditions but to drive the electrolysis at a practical rate requires higher potentials and this is conventionally measured as overpotential^9^. The difference between the equilibrium potential and the potential at practical current densities (e.g. 100 mA cm^−2^) is called polarization or overpotential (*η*). Metals such as Pt and Ru provide an excellent catalytic surface in lowering the overpotential (*η*) for the HER^10^. The most notable catalysts belong to the platinum group metals and are used for the HER, in addition, platinum group *oxides*^11^,^12^,^13^ are used for the OER. However, platinum group metals are greatly limited by its scarcity and high cost. For instance, technologies such as fuel cells cannot be scaled up because they require expensive catalysts. The most efficient electrocatalyst for the OER are metal oxide electrodes^12^,^13^ (RuO_2_ being the best^13^, IrO_2_ also shows good overpotentials^11^). Oxides present advantages over metal counterparts because they are more stable and cannot be further oxidized. Hence, the desire is to develop an inexpensive noble metal free OER catalyst.

With this as an objective, in our current paper, the use of electrolytic manganese dioxide (EMD, γ type MnO_2_) with *in-situ* addition of cobalt/molybdenum ions during electrodeposition (termed modified EMD) has been investigated as a potential catalyst towards the OER. The modified EMD significantly lowers the overpotential of the parent EMD material. Another objective of this work is to investigate the reasons for the decrease in overpotential found with these electrodes while varying the Co content in the bath solution. To the best of our knowledge, modified EMD (MEMD) materials with Co as an additive have not been reported previously. Electrolytic manganese dioxides are a well-researched area in energy storage^14^ as well as for catalysts to suppress the chlorine evolution reaction^15^,^16^,^17^,^18^,^19^ in seawater electrolysis. These studies support the concept of this material for electrochemical applications.

EMD is brittle and adding molybdenum has been found to improve its adherence to the substrate^15^, ^16^, ^17^, ^18^, ^19^. Besides molybdenum, a range of other additives in the electrolytic bath have also been investigated^15^, ^16^, ^17^, ^18^, ^19^ in the range of 3 to 9 mM. However, the cobalt additive in EMD is little known or not well established and we have used this additive in the range of 3 to 15 mM. The role of cobalt as an additive in MnO_2_ has different advantages, it lowers the water content which increases the electrical conductivity of the modified EMD, in addition, it makes the material to be energy efficient toward the OER and this is discussed in the current study. The combination of molybdenum and the extent of higher amount of cobalt in the bath, appears to be beneficial for the OER as determined in the present paper. In the design of catalysts for ammonia synthesis, the combination of Co/Mo has been experimentally-theoretically reconciled^20^ and found to outperform ruthenium (platinum group element). The current novel trend for catalyst design is the combination at the molecular scale of elements (e.g. Mo and Co) which show low and high bond strength with reaction intermediates, to generate intermediate bond strength. This is the region where catalysts operate more efficiently^20^.

In one of our previous works, we have reported MEMD for the OER in alkaline medium^21^, where a range of doping elements were investigated. The reported doping elements enhanced the overpotential at a current density of 100 mA cm^−2^. In the current study, these developed MEMD materials showed a competitive overpotential when used in acid medium, as opposed to our previous work reported for alkaline medium^21^. It should be noted that the overpotential at a current density of (*η*_10_) 10 mA cm^−2^ has recently received more attention as it is believed to be a representative variable for solar water splitting devices. However, we have used the conventional *η*_100_^9^ and found the results to be promising. The significance of this work lies in the technological improvement in electrode performance when acid medium is the electrolyte.

## Materials and Methods

### Electrode preparation

The electroplating station consisted of the plating solution, a power supply and electrodes; one 70 × 50 × 1 mm 316 stainless steel sheet, and one 20 × 10 × 1 mm IrO_2_/Ti sheet (DSA^®^ acquired from AMAC Corrosion Co.) with one face insulated by ferro-lacquer, as counter and working electrodes, respectively. An internal half-cell vessel connecting the solutions by a Nafion membrane was used. The electrolytic solutions used in the preparation of modified EMDs are tabulated in Table 1. The samples were electrodeposited at *i* = 10 mA cm^−2^. The electrodeposited samples were dried at 100 ^o^C in an oven for a period of 1 hour in an air atmosphere and used for all physico-chemical and electrochemical measurements.

### Polarization measurements

The polarization curve was performed on a three electrode set up, remotely controlled by a BioLogic VSP potentiostat. The reference (RE) and counter electrode (CE) were Ag|AgCl in a satd. KCl solution and a platinized platinum sheet with the dimensions of 10 × 10 × 1 mm, respectively. The potential of the Ag|AgCl satd. KCl electrode was taken as 197 mV vs. SHE. Modified EMD based electrodes were made anodic in 2 M H_2_SO_4_ solutions in the OER regime. The scan rate was 0.1 mV s^−1^ and the scan range was set arbitrarily from low to high current density in the range of 1 to 100 mA cm^−2^. The *IR*_drop_ compensation was carried out by the current interruption method. Finally, the geometric area of the coated sample was used in the determination of current densities and all the tests were done at 25 ^o^C. The time dependent current density experiment (under static potential) was done in sequence after cyclic voltammetry with the same set up. A chosen voltage was kept constant for a specific period of time and the current response was monitored.

### Electrochemical impedance spectroscopy (EIS)

EIS consisted of 3 consecutive scans over the frequency range from 10 kHz to 0.05 Hz performed sequentially at potentials in the OER regime. The ac sinusoidal voltage applied had amplitude of 5 mV at 25 ^o^C. A fit for purpose argental satd. KCl was used as reference electrode for this test.

### Physico-chemical Characterization

The crystallinity of the modified EMD deposits was examined by a Siemens X-ray diffractometer with Philips Co-Kα radiation (1.7902 Å). X-ray photoelectron spectroscopy (XPS) was used to examine the chemical structures in the deposits with a Thermo Multilab 2000. Carbon, C (1 s), was used as the reference spectrum. Finally, the morphology of the deposits was determined by scanning electron microscopy (SEM) equipped with energy dispersive analysis (EDS) for qualitative elemental analysis using two JEOL instruments, models JSM-7000 F and JSM-6000, respectively.

## Results and Discussions

### Electrochemical characterization of modified EMD samples

#### Tafel plots

**Figure 1:**
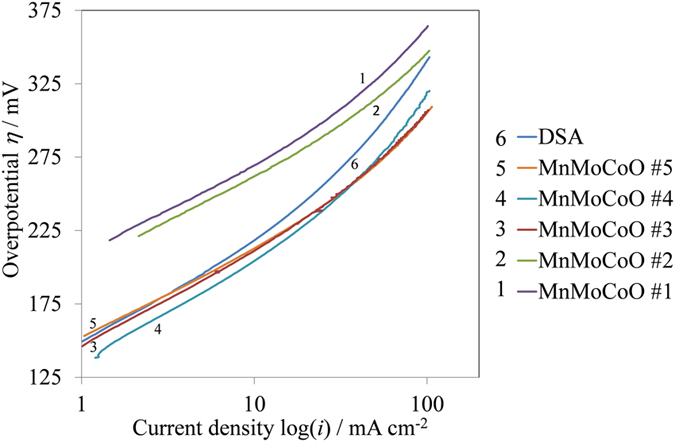
Tafel plot of the modified EMD samples in 2 M H_2_SO_4_ solution.

Figure 1 shows the Tafel plot of the modified EMD samples with Co and Mo as additives (MnMoCoO) for the OER in 2 M H_2_SO_4_ solution. For all the samples tested, the Tafel lines show one slope close to ~60 mV dec^−1^ in the low current density region and another slope at around ~115 mV dec^−1^ in the high current density region. In the supporting information, there is the theory which explains the OER mechanism steps that reconcile the different Tafel slopes found. A change in the magnitude of the Tafel slope indicates different rate determining steps (rds) which can be related to those proposed by different sources^10^,^11^. In the OER region the electrodes with varying Co content exhibit different values of overpotential. Table 2 shows the electrochemical parameters of all the tested samples. Among the modified samples studied, the sample with the highest cobalt concentration labelled #5 (i.e. MnMoCoO #5) exhibits the best overpotential (i.e. 305 mV) when compared to that from DSA^®^ at 100 mA cm^−2^ (i.e. 341 mV). DSA^®^ has been reported to have similar overpotentials (*η*) and Tafel slopes (*b*) to those determined by other research groups^11^,^22^. Hu *et al.* reported^22^ that the oxygen evolution reaction on IrO_2_ based DSA^®^ electrodes to have Tafel slopes of 59 mV dec^−1^ and 130 mV dec^−1^ at low and high overpotentials, respectively. Similar slopes have been determined. At low overpotentials the rate determining step (rds) is that described by Eqs. S-4 and S-5 (in the supporting information). For high overpotentials the rds is described by the Eq. S-1 as it yields a slope of 120 mV dec^−1^.

**Figure 2:**
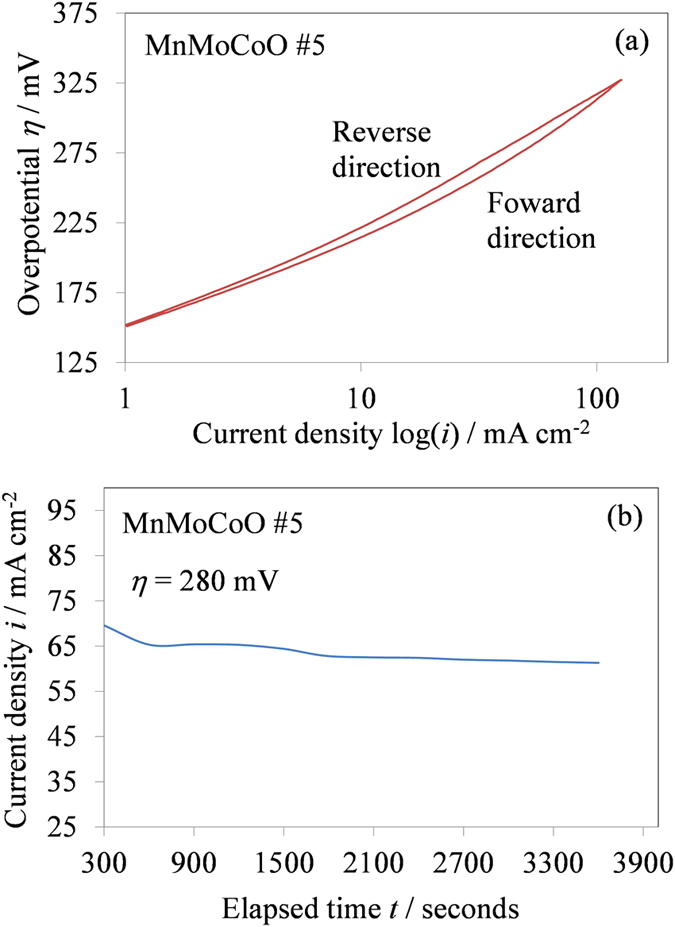
(**a**) Cyclic voltammetry curve for oxygen evolution (**b**) Time-dependent current density curve under the constant overpotential *η* = 280 mV for the MnMoCoO #5 sample (best energy efficient sample) in 2 M H_2_SO_4_ solution.

The performance characteristics of the MnMoCoO samples have been substantially improved by adding CoSO_4_ in the plating solution (see Fig. 1 and Table 2). In addition, for the electrode (MnMoCoO #5), current-potential curves were recorded in the forward and backward directions without interruption (Fig. 2a). However, a slight deviation from linearity at high overpotentials is observed. This deviation, could be attributed to ohmic drop. A steady state current potential curve for oxygen evolution has been performed for the best energy efficient sample labeled #5 (Fig. 2b). This potential-static holding has also been undertaken to evaluate the stability of the modified EMD electrode. At a chosen constant overpotential (*η* = 280 mV) the observed current density over a period of time is found to be constant.

The overpotential of the modified EMD samples in Table 2 for acid electrolyte is found to be superior to those reported from our previous work^21^ in alkaline electrolyte. A change in the electrolyte, yields different types of adsorbed reaction intermediates (i.e. Eqs. S-1 to S-5 in the supporting information). The bond strength between the catalyst surface and an adsorbed reaction intermediate, changes as a function of the adsorbate chemical structure and this could be the reason of the changes seen in the results for the overpotential found in the OER in different electrolytes. In this respect, other variables such as the exchange current density (*i*_o_) and charge transfer resistance (*R*_ct_) (*R*_ct_ is explained in the supporting information) do support the results found by Tafel slopes in Fig. 1. These variables (*i*_o_, *R*_ct_) from the MEMD samples show better values to those from DSA^®^ (see Table 2).

#### Electrochemical impedance spectroscopy plots

**Figure 3:**
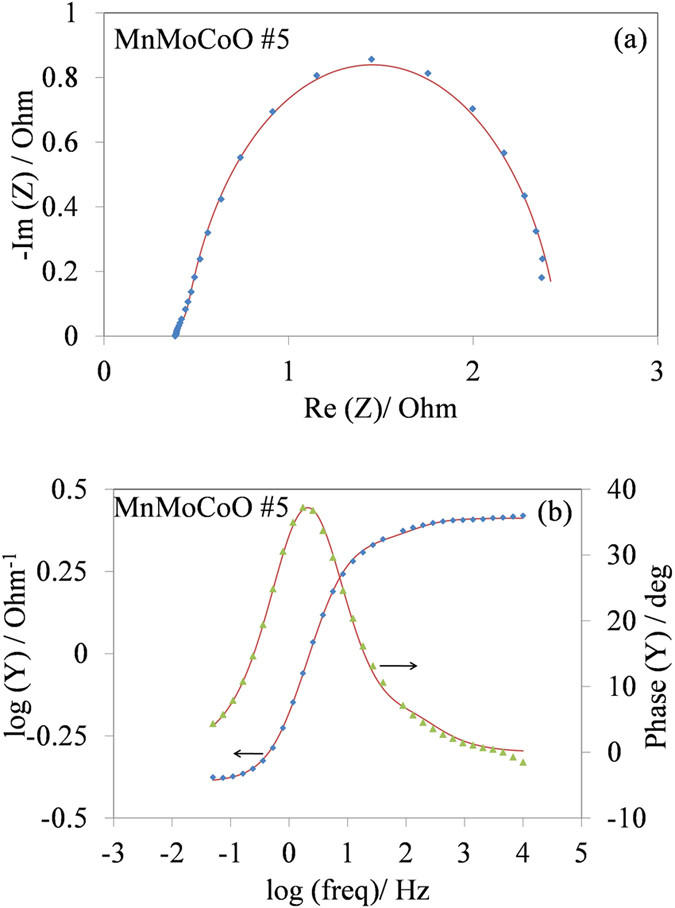
(**a**) Nyquist impedance and (**b**) Bode admittance diagrams of the MnMoCoO #5 sample at *η* *=* 245 mV in 2 M H_2_SO_4_ solution. Continuous lines represent the fitted curve by the 1CPE model, experimental data as symbols.

The equivalent circuit selected to model the EIS data is the 1CPE model^21^ (shown in Fig. S-1). Figure 3(a) shows the Nyquist impedance and 3(b) Bode admittance diagrams of the best performed sample (i.e. MnMoCoO #5) to illustrate intermediate data processing results. The curves shown in Fig. 3 are representative for all the MnMoCoO samples studied, which exhibit one capacitive loop that has been interpreted to be related to kinetic control^23^,^24^,^25^. No other capacitive or inductive loops corresponding to mass transfer control or oxide impedance are identified. Variables of importance from the 1CPE model are the *CPE*_dl_ and *R*_ct_, as they provide direct information about the roughness factor and energy efficiency. The charge transfer resistance (*R*_ct_) was determined at a current density of 10 mA cm^−2^ and this variable is close to that of the DSA^®^ sample. The results are tabulated in Table 2. *R*_ct_ is believed to directly correlate to overpotentials for the OER, as oppose to a high roughness factor, which does not necessarily means that the overpotential (*η*) will improve. Average roughness factors (*r*_f_) have been determined by Eq. S-6 and the corresponding values are tabulated in Table 2. Roughness factors have been determined to be in the same order of magnitude for all the samples, including the DSA^®^.

### Physico-chemical characterization of modified EMD samples

#### X-ray diffraction spectra

**Figure 4:**
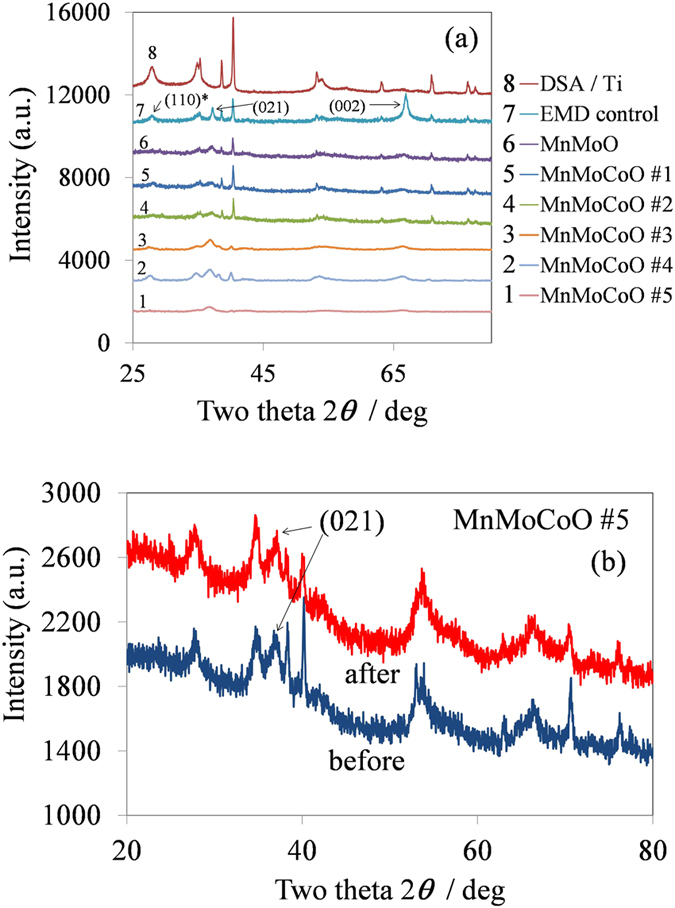
(**a**) X-ray diffraction patterns (XRD) of the modified EMD samples. (**b**) XRD of the MnMoCoO #5 sample (best energy efficient sample) before and after electrochemical measurements indicating the stability of the EMD structure.

Electrolytic manganese dioxide (EMDs) in general are mainly composed of γ-MnO_2_ phase and to a small extent by ε-MnO_2_^26^,^27^. γ-MnO_2_ is composed of pyrolusite and ramsdellite structures, they both possess a 110 diffraction line. EMDs have the 021 diffraction peak with the highest intensity followed by the 110 peak^27^. Figure 4a shows the X-ray diffraction spectra of the modified EMD samples. The control sample and substrate are also included for a comparison to study the degree of changes in the crystallinity. Three diffraction peaks with the reflections (021), (002) and (110) are identified for the standard (control) EMD material (in Fig. 4a) which does not contain the cobalt/molybdenum additives. The 110 peak in the EMD control sample is represented as 110* as the substrate exhibits a peak at the same region, this peak has not been considered for further the analysis.

For the samples with increasing cobalt additive, labeled #1 to #5 (#5 being the sample with the highest addition of Co, for details refer to Table 1) the XRD spectra show similar peaks to that of the control sample. As the content of cobalt in the EMD solution increases, the (002) peak intensity decreases. However, the (021) peak remains but becomes broad. The observed changes in the crystallinity of the samples could be one of the beneficial factors towards improved overpotential for oxygen generation. To study the chemical stability of the MEMD samples, the sample #5 is selected (best energy efficient sample towards the OER). We have performed XRD analysis before and after electrochemical measurements to determine any changes in the crystalline structure. Figure 4b shows the XRD spectra of the MnMoCoO #5 sample, before and after electrochemical measurement. As observed from the XRD (Fig. 4b) the diffraction patterns are almost identical, indicating no changes in the crystalline structure. This confirms that modified EMD is chemically stable in the acid OER regime.

#### X-ray photoelectron spectroscopy (XPS) spectra

The γ-MnO_2_ (EMD) can be described by the chemical formula^28^,^29^,^30^ as:
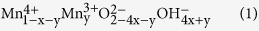
Where *x* is the cation vacancy fraction and *y* is the fraction of Mn^3+^ ions, replacing Mn^4+^ in the manganese sub-lattice. EMDs have typical values of *x* and *y* as follows: *x* ≈ 0.06 and *y* ≈ 0.075. This means that Mn^4+^ should be the dominant specie in EMDs.

**Figure 5:**
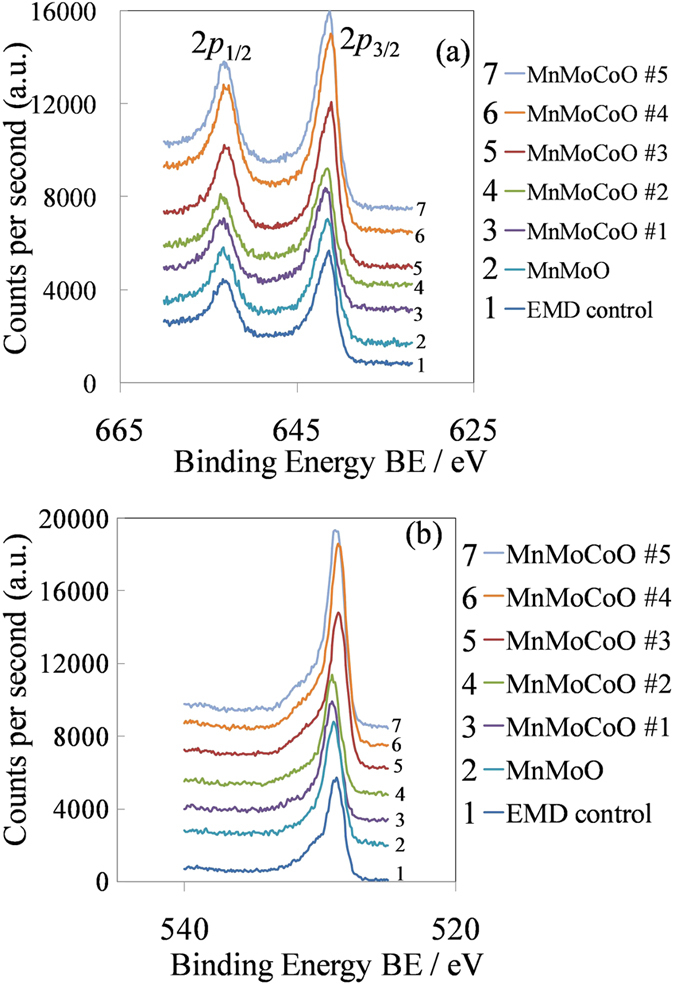
XPS spectra of the modified EMD samples. (**a**) Mn(2*p*) and (**b**) O(1*s*) spectra. A standard (control) EMD sample has been included as number 1.

Figure 5 shows the X-ray photoelectron spectroscopy (XPS) of Mn(2*p*) and O(1*s*) for the modified EMD samples, including the control sample. Figure 5a shows two peaks in the region where manganese (Mn) species are expected, these peaks correspond to the Mn(2*p*_1/2_ and 2*p*_3/2_) spin orbit split components. Comparing the XPS (Fig. 5a) results from the modified EMD samples to the standard (control) sample, it can be seen that all the XPS spectra of the samples are similar. This result suggests that the ratio of Mn^4+^ to Mn^3+^ in the MEMD is constant despite of increasing the concentration of Co^2+^ ion in the plating solution.

Figure 5b shows the O(1*s*) spectra of the samples. Considering the O(1*s*) peak parameters from Manganite^31^. The O^2−^, OH^−^ and H_2_O peaks are found at 529.6, 530.8 and 531.6 eV, respectively (O^2−^, OH^−^ and H_2_O are structural species). Comparing the O(1*s*) XPS spectra of the samples to the EMD control sample, it can be seen that the EMD control spectra has a shoulder in the region of structural water, other samples do not show this shoulder. In addition, the more Co^2+^ ion in the plating solution the more intense the O(1*s*) peak becomes. One could interpret these results as a decrease in water content that would increase the structural oxygen content in the modified EMD coating. The presence of additives (i.e. Co^2+^ ion) in the plating solution could be suppressing structural water formation during electrodeposition. These results indicate a lower concentration of structural water in the samples than that from the control EMD, which is beneficial for catalytic activity as it increases electrical conductivity and the quantity of active sites.

**Figure 6:**
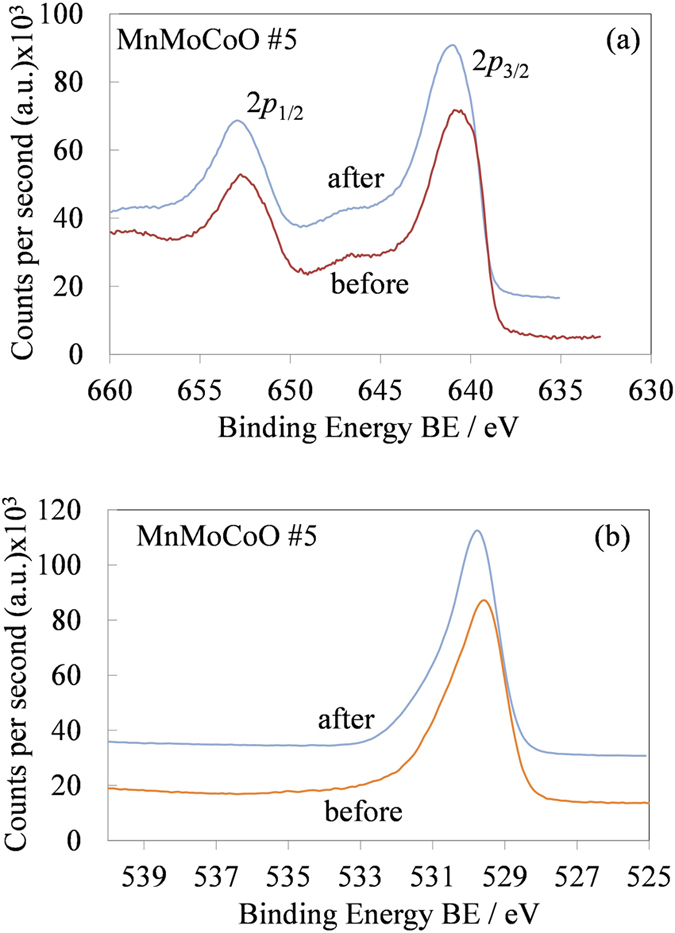
XPS spectra of the MnMoCoO #5 sample before and after cyclic voltammetry. Mn(2*p*) and O(1*s*) labeled as (**a**) and (**b**), respectively.

An additional figure has been included to analyze the chemical stability of the MEMD under the electrochemical test conditions. Figure 6 in combination with Fig. 4b, show the chemical structure of the MnMoCoO #5 sample, before and after cyclic voltammetry. Figures 6 and 4b indicate that the sample #5 is stable for the OER in acid medium.

#### Micrographs and elemental analysis FE-SEM/EDS

**Figure 7:**
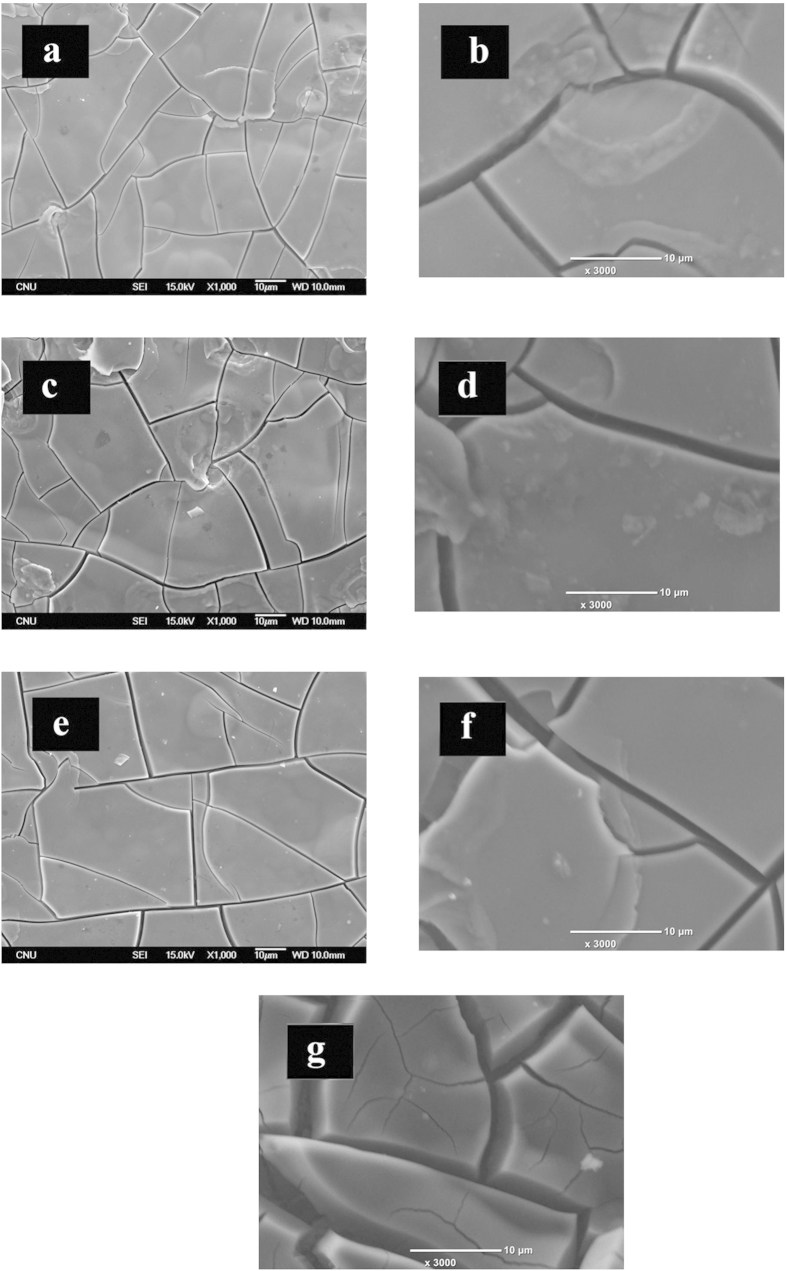
SEM imaging of the modified EMD samples. (**a,c,e**) refers to MnMoCoO #3, MnMoCoO #4 and MnMoCoO #5 (low magnification) and (b, d and f) refers to the respective samples at high magnification. Fig. 8(g) refers to MnMoCoO #5 after electrochemical measurement showing a similar morphology to that of Fig. 8(f).

Figure 7 shows the plan micrograph view of the modified EMD samples (DSA^®^ is shown^21^) at low and high magnification. These results show that all EMD based samples have a structure similar to that of dry mud. SEM results show an increase of the dry mud polygon size when the Co^2+^ content in the plating solution is increased when compared to the standard EMD. The SEM micrographs do not show a clear trend about the polygon average area enclosed by cracks as a function of Co^2+^ content in the plating solution. Bigger polygon sizes would decrease the area of the cracks and surface, this should decrease the roughness factor and hence increase the overpotential. Figure 7g also compares the surface morphology of the MnMoCoO #5 after electrochemical measurements. It is visualized from the micrograph that the morphology is quite similar, indicating that the sample is mechanically stable.

Considering the analysis from XPS and SEM/EDS (Table 3), it is found that the more Co^2+^ ion content in the plating solution, the lower the water content in the modified EMD electrodeposited samples. It is believed that water in the modified EMD sample does not become an active site, and its presence lowers the quantity or energy efficiency of the active sites making the material less electroactive. The electrochemical tests (i.e. Tafel plots and EIS) are closely related to electroactive area which does not have to be the same as the total area (i.e. true area). Water takes space of the surface of the electrode material, which can make the *R*_ct_ higher or decrease *r*_f_ depending on whether water becomes or not an active site. It can be seen that Co^2+^ content in the solution influences the composition of the coating. The higher the Co^2+^ content the less oxygen is found in the coating and this is in agreement with the results found by XPS, where water content in the modified EMD coating is lowered by Co^2+^ in solution. This is beneficial as the electrical conductivity of the coating is inversely proportional to water content.

## Conclusions

This study suggests that the Co/Mo addition in EMD electrodes played a crucial role. Modified electrolytic manganese dioxide shows a competitive overpotential (305 mV) to that of traditional high-cost DSA^®^ electrodes (341 mV) in acid OER at 100 mA cm^−2^ and this is supported by electrochemical measurements. The Co^2+^ ion additive directly influences the structure of MnO_2_ thus lowering the overpotential for the OER. Among the samples studied in varying the cobalt content, the MnMoCoO #5 sample, is found to be the best in terms of energy efficiency in acid OER than DSA^®^. The proposed explanation about the lowering of the OER overpotential found in the modified EMD samples can be related to improved combinations of charge transfer resistance and roughness factor. In particular, the charge transfer resistance correlates directly to overpotentials. XPS and EDS show that lowering the water content in the coating increases electrical conductivity, which saves energy.

## Additional Information

**How to cite this article**: Delgado, D. *et al.* Co/Mo bimetallic addition to electrolytic manganese dioxide for oxygen generation in acid medium. *Sci. Rep.* **5**, 15208; doi: 10.1038/srep15208 (2015).

## Supplementary Material

Supplementary Information

## Figures and Tables

**Table 1:** Electrolytic solutions used in the development of anodes.

Sample	Temperature (^o^C)	Time (min)	Reagents (Analytical grade)	Concentration g l^−1^	References
MnMoO and MnMoCoO # 1/2/3/4/5	90	20	MnSO_4_∙5 H_2_O	33.802	16,17,18,19
			Na_2_MoO_4_ 2 H_2_O	0.726	
			H_2_SO_4_	42.5	
			CoSO_4_∙7 H_2_O	0/0.843/1.687/3.795/5.060/6.325	

**Table 2:** Experimental electrochemical parameters of the samples for the OER in 2 M H_2_SO_4_.

Sample ID	*b* (mV dec^−1^)		*η*_100_(mV)	-log(*i*_o_)* (mA cm^−2^)	*R*_ct_* at *η*_10_(Ω cm^2^)	*r*_f_
	low *η*	high *η*				
MnMoCoO #5	59	104	305	3.34	0.290	429
MnMoCoO #4	65	121	319	2.68	0.256	630
MnMoCoO #3	62	109	306	3.76	0.475	379
MnMoCoO #2	61	114	346	3.31	0.191	645
MnMoCoO #1	61	131	364	3.42	0.315	394
DSA^®^	51	129	341	3.87	0.267	388

^*^Rounded up values based on their standard deviations.

**Table 3:** Elemental dispersive analysis (EDS) by mass of the modified EMD samples.

Sample ID	Concentration by mass (%)			
	O	Mn	Mo	Co*
MnMoO	53.28	32.10	14.62	—
MnMoCoO #1	25.77	51.77	22.46	0.00
MnMoCoO #2	12.88	63.27	20.32	3.53
MnMoCoO #3	16.43	61.58	21.71	0.28
MnMoCoO #4	7.35	79.72	12.64	0.29
MnMoCoO #5	5.65	81.25	12.77	0.33

^*^FE-SEM EDS did not detect Cobalt.
