# Supplementary material for: Co/Mo bimetallic addition to electrolytic manganese dioxide for oxygen generation in acid medium
Source: Sci Rep. 2015 Oct 15;5:15208. doi: 10.1038/srep15208 (PMC4606729; doi:10.1038/srep15208)
Supplement: Supplementary Information [file srep15208-s1.pdf]

Co/Mo bimetallic addition to electrolytic manganese dioxide for oxygen generation in acid medium

Dario Delgado[1,*], Manickam Minakshi[1], Justin McGinnity[1], Dong-Jin Kim[2]

[1]School of Engineering and Information Technology, Murdoch University, Murdoch, Australia

[2]Mineral Resources Research Division, Korea Institute of Geoscience and Mineral Resources, Daejeon, South Korea

Supporting information for publication

Theory

Delgado *et al*[1] showed different OER mechanisms in one of his previous works, however, the one selected to fit the data is that used by Da Silva *et al*[2]. This mechanism path is described by the following steps (M refers to the active site):

$$\mathrm{M} + \mathrm{H_2O} \rightarrow \mathrm{M} - \mathrm{OH_{ads}} + \mathrm{H^+} + \mathrm{e^-} \qquad \text{(S-1)}$$

$$\mathrm{M} - \mathrm{OH_{ads}} \rightarrow \mathrm{M} - \mathrm{O_{ads}} + \mathrm{H^+} + \mathrm{e^-} \qquad \text{(S-2)}$$

$$\mathrm{M} - \mathrm{O_{ads}} \rightarrow \mathrm{M} + \frac{1}{2}\mathrm{O_2} \qquad \text{(S-3)}$$

Hu[3] *et al* reported a Tafel slope of 59 mV dec^{-1} and 130 mV dec^{-1} for the OER on DSA® in acid media. He employed the mechanism given in Eqs. S-1 to S-3 to resolve EIS data at low overpotentials. However, a modification to the first OER step (i.e. Eq. S-1) has been done by dividing it into two sub-reactions[3] to account for a Tafel slope of 59 mV dec^{-1}:

$$\mathrm{M} + \mathrm{H_2O} \rightarrow \mathrm{M} - \mathrm{OH^*_{ads}} + \mathrm{H^+} + \mathrm{e^-} \qquad \text{(S-4)}$$

$$\mathrm{M} - \mathrm{OH^*_{ads}} \rightarrow \mathrm{M} - \mathrm{OH_{ads}} \qquad \text{(S-5)}$$

Where $\mathrm{M} - \mathrm{OH^*_{ads}}$ and $\mathrm{M} - \mathrm{OH_{ads}}$ are adsorption intermediates with the same chemical structure but different energy states. By including O_{ads}, there are two different chemical structural adsorbed species. Similar mechanism has been proposed by other authors[2,4]. Hu *et*

al[3] experimentally showed that the coverage of O_{ads} is substantially lower than OH^*_{ads} and OH_{ads} and close to zero. Hu *et al*[3] modeled his EIS data with a modified 1CPE circuit as shown in Fig. S-1. These results support the use of an equivalent electrical circuit for one adsorbate. Lasia[5] analytically derives the electrochemical impedance for one adsorbate reactions, commonly known as 1CPE; this model is modified by Hu *et al*[3] to account for the oxide layer. Another model is available for one adsorbate reaction but this is mainly used for porous surfaces[6], like Raney nickel based electrodes. This model is commonly known as 2CPE. Fig. S-1 shows the commonly employed models for one adsorbate reactions.

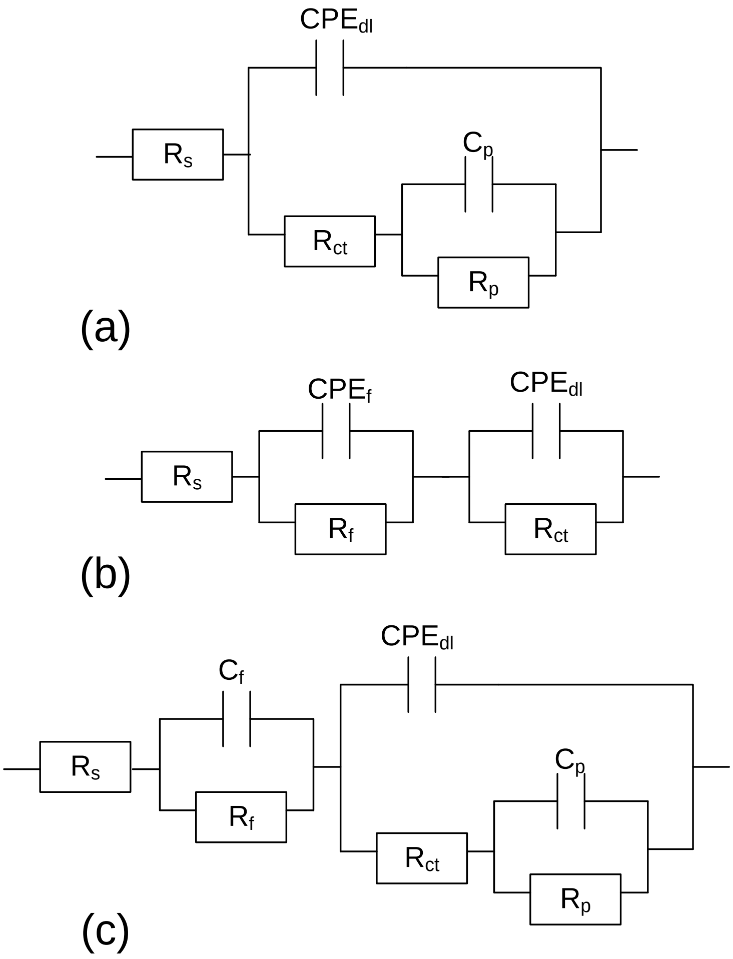

Figure S-1 Equivalent circuits for reactions with one adsorbate (a) model based on mechanism known as 1CPE; (b) model for porous surfaces known as 2CPE ; (c) modified 1CPE model to account for the impedance of an oxide layer.

All the equivalent circuits shown in Fig. S-1 have elements in common. The solution resistance is represented by R_s. The double layer capacitance and charge transfer resistance are labeled CPE_{dl} and R_{ct}, respectively. C_p and R_p are capacity and resistance, which are dependent on the rate constant values of the mechanism steps. The combination of C_f and R_f is used to represent other characteristics of the electrode surface such as porosity and oxide layer impedance, depending on the equivalent model. Electrochemical capacitances in general are represented by constant phase elements (CPE) because the surface where the reaction takes place is irregular, introducing non-ideal effects.

The double layer capacitance is used to determine the area of the electrode that participates in the reaction. In this respect, Eq. S-6 determines the average double layer capacitance for one adsorbed species reactions. The parameter T is related to the double layer capacitance and $T = C_{dl}$ when $\phi = 1$[7]. Combining this information with an estimated[3] C_{dl} of 60 µF cm^{-2} for a smooth metallic electrode, one can determine a ratio between this value and the experimentally determined C_{dl} to obtain a roughness factor (r_f).

$$T = C_{dl}{}^{\phi}(R_s^{-1} + R_{ct}^{-1})^{1-\phi} \qquad \text{(S-6)}$$

References

1 Delgado, D., Hefter, G. & Minakshi, M. in *Alternative Energies* Vol. 34 (ed German Ferreira) 141-161 (Springer, 2013).

2 Da Silva L.M., Boodts J.F.C. & L.A., D. F. Oxygen Evolution at RuO_2(x)+Co_3O_4(1-x) Electrodes from Acid Solution. *Electrochim. Acta.* **46**, 1369 (2000).

3 Ji-Ming Hu, Jian-Qing Zhang & Cao, C.-N. Oxygen Evolution Reaction on IrO_2-based DSA® Type Electrodes: Kinetics Analysis of Tafel Lines and EIS. . *Int. J. Hydrogen Energ.* **29**, 791-797 (2004).

4 De Faria L.A., Boodts J.F.C. & S., T. Electrocatalytic Properties of Ternary Oxide Mixtures of Composition $Ru_{0.3}Ti_{(0.7-x)}Ce_xO_2$: Oxygen Evolution from Acidic Solution *J. Appl. Electrochem.* **26**, 1195 (1996).

5 Lasia, A. in *Modern Aspects of Electrochemistry* Vol. 32 (eds B. E. Conway, J. Bockris, & R. E. White) 143-248 (Kluwer Academic/Plenum 1999).

6 Lasia, A. Impedance of Porous Electrodes. *J. Electroanal. Chem.* **397**, 27 (1995).

7 B. Losiewicz, A. Budniok, E. Rowinski, E. Lagiewka & Lasia, A. The Structure, Morphology and Electrochemical Impedance Study of the Hydrogen Evolution, Reaction on the Modified Nickel Electrodes. *Int. J. Hydrogen Energ.* **29**, 145 (2004).
